# High Broad‐Band Photoresponsivity of Mechanically Formed InSe–Graphene van der Waals Heterostructures

**DOI:** 10.1002/adma.201500889

**Published:** 2015-05-15

**Authors:** Garry W. Mudd, Simon A. Svatek, Lee Hague, Oleg Makarovsky, Zakhar R. Kudrynskyi, Christopher J. Mellor, Peter H. Beton, Laurence Eaves, Kostya S. Novoselov, Zakhar D. Kovalyuk, Evgeny E. Vdovin, Alex J. Marsden, Neil R. Wilson, Amalia Patanè

**Affiliations:** ^1^ School of Physics and AstronomyThe University of NottinghamNottinghamNG7 2RDUK; ^2^ School of Physics & AstronomyUniversity of ManchesterOxford RoadManchesterM13 9PLUK; ^3^ Institute for Problems of Materials ScienceThe National Academy of Sciences of UkraineChernivtsi58001Ukraine; ^4^ Institute of Microelectronics Technology RASChernogolovka142432Russia; ^5^ Department of PhysicsUniversity of WarwickCoventryCV4 7ALUK

**Keywords:** graphene, indium selenide, photoconductivity, van der Waals crystals

## Abstract

**High broad‐band photoresponsivity** of mechanically formed InSe–graphene van der Waals heterostructures is achieved by exploiting the broad‐band transparency of graphene, the direct bandgap of InSe, and the favorable band line up of InSe with graphene. The photoresponsivity exceeds that for other van der Waals heterostructures and the spectral response extends from the near‐infrared to the visible spectrum.

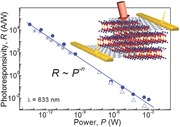

The development of van der Waals (vdW) heterostructures fabricated by mechanically stacking 2D crystals has led to the discovery of fundamental physical phenomena and to the realization of 2D functional devices ranging from sensitive photo­transistors to tunnel diodes.^1^, ^2^, ^3^, ^4^, ^5^, ^6^, ^7^, ^8^, ^9^, ^10^, ^11^, ^12^, ^13^ The electronic properties of these devices can be modified not only by careful selection of the materials within the stack, but also by adjusting the built‐in strain and relative orientation of the component crystalline layers. Among the vdW crystals, the metal chalcogenide III–VI compound, InSe, represents an exfoliable and stable semiconductor that extends the library of vdW crystals. In its bulk form, InSe has a direct bandgap *E*_g_ = 1.26 eV at *T* = 300 K, which can be increased due to quantum confinement by reducing the number of atomic layers in the crystalline sheet.^14^, ^15^, ^16^, ^17^ Recent reports of bendable photodetectors,^18^ large‐scale image sensors,^19^ electroluminescence in p–n junctions,^20^ and field‐effect transistors (FETs) with large current on/off ratios (≈10^8^) and high carrier mobilities (*μ* = 0.1 m^2^ V^−1^ s^−1^) at room temperature^21^ have demonstrated the potential of InSe for future technologies.

This burgeoning research field is still in its infancy and offers exciting opportunities for discoveries and the realization of functional devices that implement InSe in combination with other vdW crystals. Crucial to these future developments is the formation of good interfaces and Ohmic contacts to the optically active InSe layer. Devices currently used are mostly prepared from InSe with metal contacts.^14^, ^20^, ^21^, ^22^ On the other hand, the stability, flexibility, strength, high conductivity, and weak optical absorbance of graphene make this single atomic carbon layer a particularly attractive option for use as a transparent electrical contact. Furthermore, the work function of graphene can be adjusted by the electric field effect,^23^ an attractive feature for device operation and adjustable band alignment at an interface with a layered compound.

Here, we report on van der Waals graphene/InSe heterostructures formed by mechanical contact due to attractive vdW forces at the graphene/InSe interface. We exploit the broad‐band transparency of graphene and the favorable band line up of graphene with InSe to create high‐performance photo­detectors. We demonstrate vertical and planar graphene–*n*‐InSe–graphene heterostructures with a high photoresponsivity (up to ≈10^5^ A W^−1^ at *λ* = 633 nm), not yet achieved in other 2D vdW crystals, and with a spectral response that extends from the near‐infrared to the visible spectrum. The highest photoresponsivity is observed in device architectures where the InSe and graphene layers are vertically stacked and an optical window is created by overlapping the two graphene electrodes, thus enabling sensitive photodetection. These heterostructures provide innovative device architectures that enable access to fast electron speeds and high broad‐band spectral response.

Our InSe crystals were grown using the Bridgman method from a polycrystalline melt of In_1.03_Se_0.97_. The *γ*‐polytype crystal structure of InSe was probed by X‐ray diffraction (XRD) using a DRON‐3 X‐ray diffractometer in a monochromatic Cu–K_α_ radiation of wavelength *λ* = 1.5418 Å. The primitive unit cell contains three InSe layers each of which has a thickness of 8.320 Å and consists of four covalently bonded monoatomic sheets in the sequence Se‐In‐In‐Se; along the *c*‐axis, the primitive unit cell has a lattice constant of *c* = 24.961 Å and, within each *a–b* plane, atoms form hexagons with lattice parameter *a* = 4.002 Å. In its bulk form, InSe contains native donors due to In‐interstitial atoms.^24^ From Hall effect measurements on bulk InSe at *T* = 300 K, we obtain an electron density *n* ≈ 10^21^ m^−3^, an electron mobility *μ* = 0.1 m^2^ V^−1^ s^−1^, and a Fermi energy *E*_F_ ≈ 0.21 eV below the conduction band minimum.

The InSe nanosheets were prepared from the as‐grown crystals by mechanical exfoliation using adhesive tape and have thicknesses *t* that range from 20 to ≈100 nm, over which InSe retains a direct bandgap^14^, ^15^, ^16^ and a relatively high electron mobility (10^−2^–10^−1^ m^2^ V^−1^ s^−1^).^21^, ^22^ The exfoliated InSe flakes were then integrated into planar and vertical device structures incorporating graphene electrodes. We first focus on the planar devices: a graphene layer, grown on a copper substrate by low‐pressure chemical vapor deposition (CVD), was transferred onto a SiO_2_/Si substrate and patterned by electron beam lithography (EBL) into two contacts prior to the transfer of an InSe nanoflake (see the Experimental Section for details of the device fabrication). **Figure** **1**a illustrates the schematic layered structure and an optical image of a graphene–*n*‐InSe–graphene planar device structure with Au‐metal contacts on two graphene layers. The *n*‐Si layer serves as a gate electrode and the graphene layers, g_1_ and g_2_, serve as source and drain to an InSe channel of length *l* ≈ 2 μm, width *w* ≈ 10 μm, and thickness *t* ≈ 30 nm.

**Figure 1:**
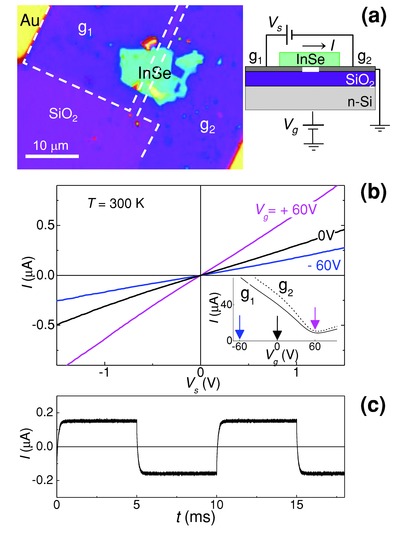
a) Optical image and schematic structure of a graphene–*n*‐InSe–graphene planar device on a gated SiO_2_/Si substrate. b) Current–voltage, *I–V*_s_, characteristics in the dark at *T* = 300 K at various gate voltages, *V*_g_. The inset shows the *V*_g_‐dependence of the current through each graphene electrode, g_1_ and g_2_. This is measured using separate Au‐contacts on each graphene layer under an applied bias of 0.1 V. c) Temporal dependence of the dark current modulated by an AC square bias *V*_s_ signal of frequency *f* = 100 Hz and amplitude of 1 V (*V*_g_ = 0 V).

As shown in Figure 1b, the current through the InSe flake has a linear dependence on the bias voltage *V*_s_ applied between the two graphene electrodes. The *I–V*_s_ characteristics are symmetric relative to negative and positive values of *V*_s_ and, for each *V*_s_, the current increases with increasing gate voltage *V*_g_. Separate Au‐contacts on each graphene layer enable us to assess their conductivity: they exhibit a linear dependence of the current on the bias voltage and a minimum conductance at around a gate voltage *V*_g_ ≈ 60 V, corresponding to p‐type doping and hole concentration of *p* ≈ 5 × 10^12^ cm^−2^, typical for CVD graphene (inset of Figure 1b).^25^ The transport characteristics of the device are reproducible and stable; also, a fast response time is observed with a cut‐off frequency of *f* ≈ 10^4^ Hz. Figure 1c shows the characteristic temporal dependence of the current modulated at a frequency of *f* = 100 Hz, with rise (*τ*_r_) and decay (*τ*_d_) times of the current of *τ*_d_ ≈ *τ*_r_ < 0.1 ms.

The linearity and symmetry of the *I–V*_s_ characteristics are preserved under optical illumination, see **Figure** **2**a. The spatially resolved photocurrent map obtained by scanning a focused laser beam across the plane of the graphene/InSe/graphene device shows that photocurrent generation occurs primarily in the InSe region of the flake between the two graphene electrodes (see the inset of Figure 2a). A broad‐band spectral response from the near‐infrared to the visible parts of the spectrum is observed under unfocussed optical illumination, see Figure 2b. The photoinduced current, Δ*I*, is weakly modulated by the gate voltage *V*_g_ and its dependence on *V*_g_ is opposite to that observed for the dark current, i.e., Δ*I* decreases with increasing *V*_g_ from −60 to +60 V. As shown in Figure 2c, the temporal dependence of Δ*I* is slower than that of the dark current with a rise time *τ*_r_ ≈ 1 ms and decay time *τ*_d_ ≈ 10 ms, which are not affected by *V*_g_.

**Figure 2:**
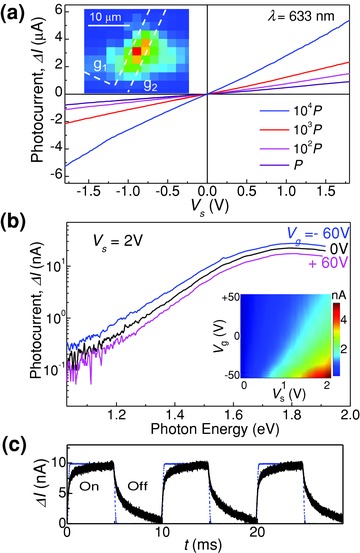
a) Photocurrent Δ*I* versus *V*_s_ at *T* = 300 K and *V*_g_ = 0 V under light illumination with a focused laser beam of power *P*, 10^2^ *P*, 10^3^ *P*, and 10^4^ *P* (*P* = 0.5 nW, *λ* = 633 nm). The inset shows the photocurrent map indicating that Δ*I* arises primarily from the InSe layer between the two graphene electrodes, g_1_ and g_2_. b) Photoconductivity spectra at *T* = 300 K and *V*_s_ = 2 V with unfocused light and power *P* ≈ 10 pW. The inset shows a color map of Δ*I* versus *V*_g_ and *V*_s_ with a laser at *λ* = 633 nm. c) Temporal dependence of the photocurrent (*V*_g_ = 0 V, *V*_s_ = 1 V, and *λ* = 633 nm). The dashed line shows the modulation of the laser beam at frequency *f* = 100 Hz.

High‐quality interfaces, crystal lattice alignment and control over the relative band alignment are essential for tuning and controlling electrical injection of carriers from an electrode into a semiconductor layer. The cleaved facets of InSe layered crystals are atomically smooth, as confirmed by our atomic force and transmission electron microscopy studies (not shown), and contain a low‐density of surface states,^26^, ^27^ which makes the surface of this material stable compared to covalent semiconductors, such as Si; this also enables the formation of abrupt heterointerfaces when InSe is combined with metals and/or other layered compounds.^20^, ^26^, ^27^ Thus, to model the InSe/graphene heterostructure we consider the band alignment taking into account the electron affinities and work functions of InSe and graphene, but neglecting the possible presence of defects and/or impurities at the interface.

As shown in **Figure** **3**a, the electron affinity of graphene (*χ*_g_ = −4.5 eV) is larger than that of bulk InSe (*χ*_InSe_ = −4.6 eV);^26^ furthermore, the graphene work function (*φ*_g_) can be increased relative to that of n‐type InSe (*φ*_InSe_ = – 4.81 eV) by applying a gate voltage. Compared to InSe, this gate effect is stronger in graphene due to the low density of states of graphene when the chemical potential is close to the Dirac point. In particular, over the range of applied gate voltages *V*_g_ from −60 to +60 V, we find that *φ*_g_ remains higher than or comparable to *φ*_InSe_, with the Fermi level increasing from ca. −0.3 eV (*φ*_g_ = −4.85 eV at *V*_g_ = −60 V) to 0 eV (*φ*_g_ = −4.5 eV at *V*_g_ = +60 V) relative to the neutrality point of the Dirac cones. Thus, we infer that at equilibrium, electrons tend to transfer from graphene to InSe where they can form an accumulation layer at the interface with graphene (Figure 3b). This alignment of the bands at each graphene/InSe interface facilitates the formation of an Ohmic contact, consistent with the linearity of the measured *I–V*_s_ (see Figure 1b).

**Figure 3:**
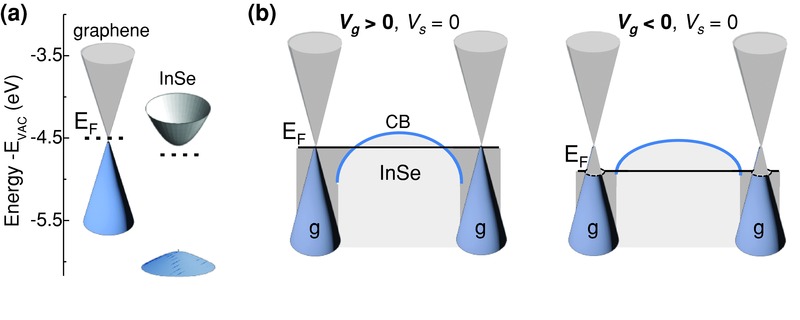
a) Energy bands for isolated graphene and InSe layers with electron affinities of *χ* = −4.5 and −4.6 eV, respectively, and a bandgap energy for InSe of *E*_g_ = 1.26 eV at 300 K. The Fermi level, *E*_F_, is shown for graphene at the neutrality point and for *n*‐InSe at 0.21 eV below the conduction band (CB) minimum. b) Band alignment at equilibrium (*V*_s_ = 0) under various applied gate voltages *V*_g_. For *V*_g_ > 0, the Fermi level of graphene raises toward the Dirac point and electrons tend to diffuse into the InSe layer; as the concentration of holes (induced by a negative gate voltage) increases, the Fermi level in graphene moves closer to that of InSe and electrons retreat from InSe.

The band alignment is supported by the opposite dependence on the applied gate bias observed for the dark current and the photocurrent. To explain the monotonous increase of the dark current with increasing *V*_g_ (Figure 1b) and the decrease observed for the photocurrent (Figure 2b), we should examine the modulation of the electronic conduction by the gate voltage. As shown in Figure 3b, for a positive gate voltage *V*_g_ > 0, the Fermi level in graphene moves upward toward the Dirac point and more electrons can diffuse into InSe, thus decreasing the effective length *l* over which the bias *V*_s_ is dropped; in contrast, for *V*_g_ < 0, the Fermi level in graphene moves to lower energies, closer to the Fermi energy of InSe, and electrons retreat from InSe thus increasing *l*. Thus for *V*_g_ < 0, the length *l* is longer than for *V*_g_ > 0; correspondingly, the dark current decreases as *V*_g_ is made more negative, but the photocurrent increases due to an increased number of carriers photogenerated over a larger length *l*. From the change of the photocurrent (ca. ±20%) over the range of *V*_g_ from −60 to +60 V, we estimate that *l* changes by ca. ±20%. This dependence was not observed in the Au–InSe–Au planar devices where the dark current and photocurrent both decrease or increase with varying the gate voltage.

Under an applied bias *V*_s_, electrons and holes that are photoexcited in InSe are swept by the electric field in opposite directions and are extracted at the graphene electrodes to generate a photocurrent Δ*I* = [*etαP*/*hv*]*τ_l_*/*τ*_t_, where *P* is the incident power, *α* is the absorption coefficient of InSe at the photon energy *hv*, *e* is the electron charge, *t* is the thickness of the InSe layer, and *τ*_l_/*τ*_t_ is the ratio of the minority carrier lifetime (*τ*_l_) and transit time (*τ*_t_) of electrons in InSe (for the derivation of Δ*I*, see Section SI, Supporting Information). Thus the photoresponsivity *R* of our device can be approximately described by *R* = Δ*I*/*P* = [*etα*/*hv*]*τ*_l_/*τ*_t_. Furthermore, we can express the external and internal quantum efficiencies as *EQE* = *R*(*hv*/*e*) = [*tα*]*τ*_l_/*τ*_t_ and *IQE* = *τ*_l_/*τ*_t_, respectively. These relations indicate that large values of *R*, *EQE*, and *IQE* can be achieved if the lifetime of the minority carriers (holes) is longer than the transit time of electrons.

**Figure** **4**a shows the measured photoresponsivity at *V*_s_ = 2 V, *V*_g_ = 0 V, and *λ* = 633 nm: *R* is strongly dependent on the optical power *P* and reaches a maximum value of *R* = 4 × 10^3^ A W^−1^ at the lowest incident power *P* = 10 pW, which corresponds to *τ*_l_/*τ*_t_ ≈ 3 × 10^5^ for *α* = 10^6^ m^−1^ at *hv* = 1.96 eV (*λ* = 633 nm) and *t* = 30 nm. From the measured values of *R*, we estimate a maximum external quantum efficiency *EQE* = *R*[*hv*/*e*] ≈ 5 × 10^3^ and a specific detectivity D* = *R*(*A*/*2eI*)^1/2^ ≈ 10^10^ m W^−1^ s^–1/2^, where *A* = 20 μm^2^ is the area of the device and *I* = 0.6 × 10^−6^ A is the dark current at *V*_s_ = 2 V and *V*_g_ = 0 V. As shown in Figure 2b, the photoresponse depends on the photon energy and we estimate that *R* and *EQE* decrease by a factor of ≈30 and 50, respectively, for *hv* decreasing from ≈2 to ≈1.3 eV.

**Figure 4:**
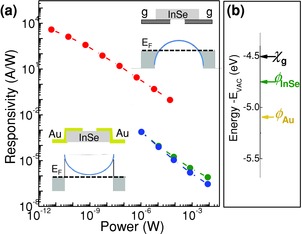
a) Photoresponsivity versus incident laser power at *T* = 300 K and *λ* = 633 nm for planar device geometries based on graphene/InSe/graphene (top) and Au/InSe/Au heterostructures (bottom) (*V*_s_ = 2 V and *V*_g_ = 0 V). The inset sketches the band alignment for the two heterostructures at *V*_s_ = 0 V. b) Work functions (*φ*) for Au and InSe, and the electron affinity (*χ*) of graphene.

The decrease of *R* with increasing *P* is analogous to the *P*‐dependence reported previously for the photoresponsivity of other graphene‐based photodetectors.^4^ According to our model, the photoresponsivity is determined by the ratio *τ*_l_/*τ*_t_. Thus the decrease of *R* with increasing *P* suggests a decrease of *τ*_l_ and/or an increase of *τ*_t_. The transit time of photoinduced carriers can be increased by the power due to enhanced scattering.^28^ Also, an increasing power can induce Auger recombination processes and increases the carrier recombination rate, thus reducing *τ*_l_. Since this behavior is not observed in devices based on thick bulk InSe flakes, we infer that Auger‐like carrier recombination on traps is enhanced in these 2D vdW crystals due to stronger Coulomb interactions. Finally, *R* decreases linearly with decreasing applied bias voltage *V*_s_, as expected from the increase in the electron transit time *τ*_t_ = *l* ^2^/*μV*_s_: for *V*_s_ = 2 V, *l* = 2 μm, and *μ* = 0.1 m^2^ V^−1^ s^−1^, we estimate a transit time *τ*_t_ ≈ 2 × 10^−11^ s and a carrier lifetime *τ*_l_ ≈ 5 × 10^−6^ s.

Our values of *R* and *EQE* for the InSe/graphene photo­detector are significantly larger (by a factor of 10^4^) than those measured in devices in which Au‐contacts replace the two graphene electrodes on the InSe flake, see Figure 4a. We attribute the lower photoresponsivity in the Au–InSe–Au heterostructure to the presence of a Schottky barrier at the Au/n‐type InSe interface (inset of Figure 4a). As shown in Figure 4b, this could form because the work function for Au (*φ*_Au_ = −5.2 eV) is lower than that for n‐InSe (*φ*_InSe_ = −4.8 eV), thus resulting in a Schottky contact at each interface of the heterostructure and in non‐linear *I*–*V*_s_ (not shown). A barrier may also form due to a defective interface between InSe and the metal contact, as reported recently by Feng et al.^22^

Our data show that the graphene electrodes enable an efficient extraction of photogenerated carriers in *n*‐InSe. Since graphene is optically transparent, high photoresponsivity can also be achieved in multilayer systems where the InSe and graphene layers are vertically stacked with the top graphene layer acting as a broad‐band optical window. These structures have the additional advantage that the separation of the graphene electrodes, determined by the thickness *t* of the InSe flake, is smaller leading to a more sensitive photodetector through a decrease of the carrier transit time. However, compared to the planar devices, for the fabrication of the vertical graphene/*n‐*InSe/graphene structure, additional process steps are required to pattern the upper graphene contact layer. Here we have developed different processing steps, corresponding to devices architectures of type A (**Figure** **5**a) and type B (Figure 5b and Figure S2, Supporting Information), which make use of exfoliated‐graphene and graphene grown by CVD, respectively (see the Experimental Section and Section S2, Supporting Information for details of the device fabrication).

**Figure 5:**
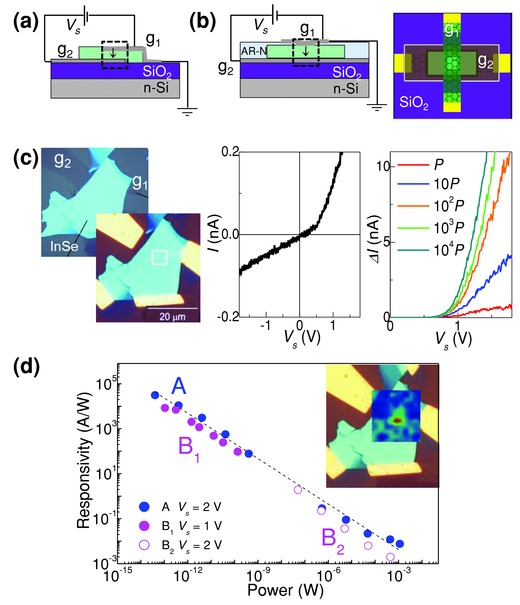
Schematic structure of graphene–*n*‐InSe–graphene vertical devices of a) type A and b) type B. c) Left: Optical images of device A showing the heterostructure before and after deposition of the Au‐contacts. The graphene layers, g_1_ and g_2_, are separated by an InSe flake of thickness *t* = 27 nm and overlap over a small region shown by the square. Right: Dark current, *I*, and photocurrent, Δ*I*, versus *V*_s_ at *T* = 300 K for device A. The photocurrent is measured with a laser beam of power *P*, 10*P*, 10^2^ *P*, 10^3^ *P*, and 10^4^P (*P* = 40 fW, *λ* = 633 nm, *T* = 300 K). d) Photo­responsivity versus laser power at *T* = 300 K and *λ* = 633 nm for device A (blue) and devices of type B (magenta). Devices B_1_ and B_2_ are based on InSe flakes of thickness *t* = 130 and 80 nm, respectively. The line is a fit to data for device A by an empirical power law, i.e., *R* ≈*P^−n^*, where *n* = 2/3. The inset shows a photocurrent map superimposed onto the optical image of device A.

Figure 5c shows optical images of device A. Here the top‐(g_1_) and bottom‐graphene (g_2_) layers overlap an InSe nanoflake of thickness *t* = 27 nm. The size of the optical window on the top surface of the InSe is approximately 4 μm × 4 μm (see square in Figure 5c) and corresponds to the region of the InSe flake whose optical excitation generates a photocurrent. As shown in Figure 5c, the dependence of the dark current, *I*, on the applied bias *V_s_* is non‐linear. This behavior, which is different from that observed for the planar device (Figure 2), may arise from a spurious contact resistance at the InSe/graphene interface caused by surface defects or residual organic material introduced during the fabrication process (see discussion in the Experimental Section).

Devices of type A and B exhibit a stable and reproducible photoresponsivity (Figure 5d) with values of *R* of up to 10^5^ A W^−1^ at *λ* = 633 nm and response to light at powers *P* as low as 10^−14^ W over an area of the InSe flake of ≈16 μm^2^ for device A (see photocurrent map in the inset of Figure 5d). From this value of *R*, we estimate an external quantum efficiency *EQE* = *R*[*hv*/*e*] ≈ 10^5^ and a specific detectivity D* = *R*(*A*/*2eI*)^1/2^ ≈ 10^13^ m W^−1^ s^–1/2^, where *A* = 16 μm^2^ is the area of the device and *I* = 0.4 × 10^−9^ A is the dark current at *V*_s_ = 2 V and *V*_g_ = 0 V. Finally, we note that a power law relation of the form *R* = *CP^−n^*, with *n* ≈ 0.7 where *C* is a constant, provides a good empirical fit to data of *R* for both vertical (see dashed line in Figure 5d) and planar heterostructures.

Overall, the photoresponsivity measured in our planar and vertical devices is significantly larger than that reported for photodetectors based on graphene and/or vdW 2D crystals at similar wavelengths and laser powers, which do not exceed values of 10^3^ A W^−1^ (see review by Feng et al.^22^). We attribute the enhanced photoresponsivity and detectivity of these devices to the favorable alignment of the bands at the graphene/InSe interface. In particular, our devices are based on InSe flakes that are thicker (>20 nm) than other 2D vdW crystals, e.g., MoS_2_, WS_2_ reported recently, and InSe remains a direct‐gap semiconductor down to thicknesses of a few nanometers. In contrast, transition metal dichalcogenides, such as MoS_2_, have a direct‐bandgap only in the monolayer form.^29^ Also, we note that although a higher photoresponsivity (10^7^ A W^−1^) has been achieved in phototransistors based on graphene and colloidal nanocrystals,^30^ the performance of such hybrid photodetectors tends to be compromised by a slow (≈1s) optical response due to the slow escape rate of photogenerated charges from the strongly confined nanocrystals. In contrast, for our 2D structures, the mechanism responsible for the photoresponse does not rely on a charge trapping effect as light generates free carriers; in addition, the extraction of both electrons and holes at the graphene electrodes is facilitated by a low potential barrier at each InSe/graphene interface. These features of our heterostructures enable relatively fast transit times for carriers and modulation of the dark and photocurrent at millisecond time scales.

In summary, our findings demonstrate that mechanically formed heterojunctions of InSe and graphene have optical and electrical properties with potential for applications in optoelectronics. Our innovative fabrication methods could be extended to other material systems and device architectures. It should be possible to achieve different band alignments and potential profiles by combining InSe with other layered semiconductors, and by selecting the doping, n‐ or p‐type, of the component layers, which is difficult to achieve in other van der Waals 2D crystals. For example, further developments include systems in which the InSe layer is replaced by multiple bandgap heterostructures, such as InSe/GaSe or InSe/hBN junctions and quantum wells. This type of vertical heterostructure architecture would enable fast electron speeds; photonic applications are also attractive due to the potential to access an even wider spectral range than that achieved in our current devices.

## Experimental Section

Images of the InSe flake and graphene topography were acquired by atomic force microscopy (AFM) in noncontact mode under ambient conditions. The experimental setup for the photoconductivity maps comprised a He–Ne laser (*λ* = 633 nm), an *XY* linear positioning stage and an optical confocal microscope system. The laser beam was focused to a diameter *d* ≈ 1 μm using a 100× objective and the maps were measured at low power (*P* < 0.1 mW) to avoid lattice heating.

For the measurement of the photocurrent spectra (Figure 2b), light from a 250 W quartz halogen lamp was dispersed through a 0.25 m monochromator (bandwidth of ≈10 nm). Light was modulated with a mechanical chopper (frequency *f* = 7 Hz) and focused onto the device. The photocurrent signal was measured using a Stanford SR830 lock‐in amplifier (integration time constant of *t* = 3 s).

The measurements of the DC dark current and photocurrent versus the applied voltage (Figures 1b and 2a) were made using a Keithley 2400 source‐meter. For the temporal studies of the dark current (Figure 1c), we used a TTi TGA 1241 arbitrary waveform generator and a Tektronix DPO 4032 digital oscilloscope. The response of the dark current to an AC square driving signal was studied in the frequency range *f* = 10^−1^–10^5^ Hz. The temporal dynamics of the photocurrent (Figure 2c) was investigated under constant bias voltage (*V*_s_ = ±1 V) and illumination by a mechanically modulated He–Ne laser with *λ* = 633 nm, *P* ≈ 10^−5^ W cm^−2^ and frequency *f* in the range 1–400 Hz. The photocurrent signal was measured using a Tektronix DPO 4032 digital oscilloscope and a Keithley2400 was used as a DC voltage source. For these AC measurements, the device was connected in series with a 1 MΩ resistor. We measured the voltage drop across the resistor, which enabled us to measure voltage signals with a low noise level.

*Fabrication of Planar Graphene/InSe Heterostructures*: The exfoliated InSe flakes were integrated into 2D planar structures incorporating either Au or graphene contacts fabricated by EBL. In these devices, CVD‐graphene, grown on a copper substrate, was transferred to a SiO_2_/Si substrate (SiO_2_ layer thickness of 300 nm) by first coating it with poly(methyl methacryrate) (PMMA) and placing on the surface of a FeCl_3_ etchant (Transene, CE‐100) to remove the copper. The PMMA/graphene film was then placed in a HCl bath and subsequently rinsed in deionized water and then mechanically placed on the substrate, before removing the PMMA by immersion in acetone, rinsing in isopropyl alcohol and annealing in an Ar:H (95:5) mixture at 400 °C for several hours. The graphene was then patterned using electron beam lithography and etching in an Ar/O_2_ plasma and, following removal of PMMA, as described above, transferred to a N_2_ atmosphere where the InSe was exfoliated and transferred to the substrate. Au/Ti contact pads were formed to provide contacts to the graphene layers, resulting in the structures shown in Figure 1a.

*Fabrication of Vertical Graphene/InSe Heterostructures*: The fabrication of the vertical devices is more complex than for the planar devices and requires several steps. We have used two methods, referred to as methods A and B, both described below.

*Method A*: Devices of type A (Figure 5a) were formed using exfoliated graphene to form similar structures to the vertical graphene/boron nitride tunneling devices by Dean et al.^11^ In this approach the bottom monolayer graphene electrode was formed by mechanically exfoliating graphite using a low‐tack, low‐residue tape, and peeling directly onto a SiO_2_/Si substrate (SiO_2_ layer thickness of 290 nm) which had been cleaned in acetone, isopropyl alcohol and an Ar/O_2_ plasma. A suitable monolayer was then located using a microscope. The InSe flake (thickness *t* = 27 nm) and top monolayer graphene electrode were also formed by mechanical exfoliation using the low‐tack low‐residue tape, but were deposited onto PMGI (poly dimethyl glutarimide)/PMMA coated substrates for later transfer. Suitable flakes of each material were selected and isolated (using a sharp implement to score a circle in the PMMA around each flake); the layer of PMGI underneath each of the isolated flakes was then dissolved using MF‐319 developer. The flakes, along with their supporting PMMA membranes, were then floated onto the surface of a beaker of distilled water and deionized water. Each membrane was captured on a PMMA‐coated stainless‐steel washer and the washer was attached to a “transfer arm” connected to a set of micromanipulators; these micromanipulators were then used to position each flake over the previous layer(s) of the device before the membrane was brought into contact with the substrate. Finally, the membrane was cut free of the washer, baked onto the substrate at 130 °C and the PMMA was washed away in an acetone bath to leave the desired flake in place. Au/Cr contact pads (50/3 nm) were formed to provide contacts to the two graphene layers.

*Method B*: Devices of type B (Figure 5b and Section S2, Supporting Information) were fabricated using large area CVD graphene grown on copper. Following deposition of Au/Ti alignment marks on a SiO_2_/Si substrate (SiO_2_ layer thickness of 300 nm), graphene was transferred and cleaned, as described above. InSe flakes were deposited by exfoliation and a flake of thickness *t* was selected using optical microscopy (*t* = 130 and 80 nm in devices B_1_ and B_2_, respectively). The graphene layer was patterned and etched in an Ar/O_2_ plasma to leave a graphene strip, which provided a continuous connection to the (unetched) graphene under the selected InSe flake. Au/Ti contact pads (110/15 nm) were then deposited at each end of the lower contact strip. In the same step Au/Ti pads were also formed to provide contacts to the upper graphene strip, which was deposited in a later fabrication step (see below). An isolation layer formed by exposing a negative resist, AR‐N, using electron beam lithography was then formed; this covered the edges of the InSe flake and the regions of the lower graphene contact close to the flake, to avoid the formation of electrical shorts between upper and lower graphene strips. A window with an area of approximately 2 μm × 2 μm was formed in the AR‐N layer on the top surface of the InSe to facilitate the mechanical contact with the upper graphene layer. Finally, a second layer of graphene was transferred as described above; in this case the supporting PMMA film, which was added to the graphene/copper before etching in FeCl_3_, was not removed, but instead used as the resist layer in a further stage of electron beam lithography in which the upper graphene contact strip was defined by etching in an Ar/O_2_ plasma. The resulting upper contact runs at right angles to the lower strip to form a graphene/InSe/graphene vertical heterostructure and overlaps the Au/Ti contacts formed at an earlier stage of the process (see Figure S2, Supporting Information).

The non‐linearity of the *I*(*V*) characteristics of the devices A and B is possibly related to the inclusion of organic residues at the graphene/InSe interface. This may arise from polymeric layers used during the process (including the negative resist layer for type B devices); in addition the InSe layer is submerged in water during the transfer of the upper graphene contact.

## Supporting information

As a service to our authors and readers, this journal provides supporting information supplied by the authors. Such materials are peer reviewed and may be re‐organized for online delivery, but are not copy‐edited or typeset. Technical support issues arising from supporting information (other than missing files) should be addressed to the authors.

Supplementary
