# Supplementary material for: High Broad‐Band Photoresponsivity of Mechanically Formed InSe–Graphene van der Waals Heterostructures
Source: Adv Mater. 2015 May 15;27(25):3760–6. doi: 10.1002/adma.201500889 (PMC4768130; doi:10.1002/adma.201500889)
Supplement: Supplementary file 1 — Supplementary [file ADMA-27-3760-s001.pdf]

Copyright WILEY-VCH Verlag GmbH & Co. KGaA, 69469 Weinheim, Germany, 2015.

ADVANCED MATERIALS

Supporting Information

for *Adv. Mater.,* DOI: 10.1002/adma.201500889

High Broad-Band Photoresponsivity of Mechanically Formed InSe–Graphene van der Waals Heterostructures

*Garry W. Mudd, Simon A. Svatek, Lee Hague, Oleg Makarovsky, Zakhar R. Kudrynskyi, Christopher J. Mellor, Peter H. Beton, Laurence Eaves, Kostya S. Novoselov, Zakhar D. Kovalyuk, Evgeny E. Vdovin, Alex J. Marsden, Neil R. Wilson, and Amalia Patanè**

Supporting Information

S1. Photodetection in planar and vertical g-InSe-g heterostrcutures

First we consider the planar graphene-InSe-graphene heterostructure of Fig. S1a. The *n*-type InSe channel has width *w*, thickness *t*, and length *l*, with ohmic contacts across the length *l*. When light of power *P* and energy *hv* larger than the band gap energy of InSe falls on the surface *xy* of the InSe flake, electron-hole pairs are generated. The densities of photo-created electrons and holes are equal, *i.e.* $\Delta n=\Delta p$, and, at steady state, they can be expressed in terms of the minority carrier (hole) lifetime, τ_l, and the rate of carrier generation by light *G* [S1], *e.g.*

$$\Delta n = \Delta p = G\tau_l, \tag{1}$$

where $G = (t\alpha)(P/hv)(wtl)^{-1}$ and α is the absorption coefficient of InSe at the energy *hv*.

Since the mobility of electrons is much larger than that of holes, we express the photocurrent ΔI along the *x*-direction in terms of the dominant electron current, *e.g.*

$$\Delta I = \Delta n e v_d (wt), \tag{2}$$

where $v_d= l/\tau_t$ is the electron drift velocity and τ_t is the electron transit time across the length *l* of the channel. Using equations 1-2 and the relations for *G* and v_d, we express ΔI as

$$\Delta I = (et\alpha P/hv)(\tau_l/\tau_t), \tag{3}$$

from which we derive the photoresponsivity *R*

$$R=\Delta I/P= (et\alpha/ hv)(\tau_l/\tau_t). \tag{4}$$

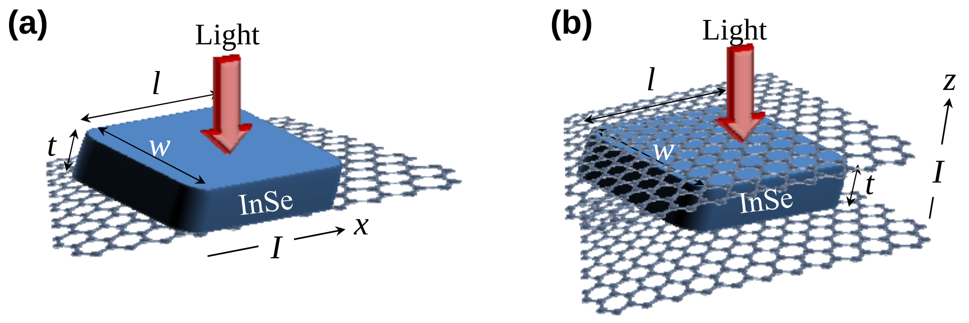

Figure S1: Schematic diagrams of a planar (a) and a vertical (b) graphene-InSe-graphene heterostructure under optical illumination.

We now consider the vertical graphene-InSe-graphene heterostructure of Figure S1b. In this case, the photocurrent ΔI is directed along the z-direction and is given by

$$\Delta I = \Delta n e v_d (wl), \qquad (5)$$

where $v_d = t/\tau_t$ and τ_t is the electron transit time across the thickness t of the InSe flake. Thus, taking into account the expression of v_d and ΔI for the vertical device geometry, we derive for ΔI and R the same expressions as those for the planar device in equations 3 and 4.

From equation 4 for R, we derive the external quantum efficiency EQE

$$EQE = R\,(h\nu/e) = (t\alpha)(\tau_l/\tau_t), \qquad (6)$$

and the internal quantum efficiency IQE

$$IQE = (\Delta I/Pt\alpha)(h\nu/e) = \tau_l/\tau_t. \qquad (7)$$

The specific detectivity D^* is defined as

$$D^* = R\,(fA)^{1/2}/I_{noise}, \qquad (8)$$

where I_{noise} is the noise current, f is the frequency bandwidth and A is the area of the channel. If we assume a dominant shot noise in the dark current I, we can rewrite equation 8 as

$$D^* = R\,(A/2eI)^{1/2}. \qquad (9)$$

Finally, we use equation 4 for R, to express D^* as

$$D^* = (t\alpha\tau_l/h\nu)(2n_o l\,\tau_t)^{-1/2}, \qquad (10)$$

for the planar device and as

$$D^* = (t\alpha\tau_l/h\nu)(2n_o t\,\tau_t)^{-1/2}. \qquad (11)$$

for the vertical device, where n_0 is the electron density in the dark.

Thus for the vertical graphene-InSe-graphene heterostructure, the smaller separation *t* between the electrodes should lead to a faster electron transit time τ_t and hence improved photodetection, *i.e.* larger values of *R*, *EQE*, *IQE*, and *D**.

[S1] S.M. Sze, Semiconductor devices, Physics and Technology, 2nd edition, pages 61-62.

S2. Fabrication of graphene/*n*-InSe/graphene heterostructures using method B

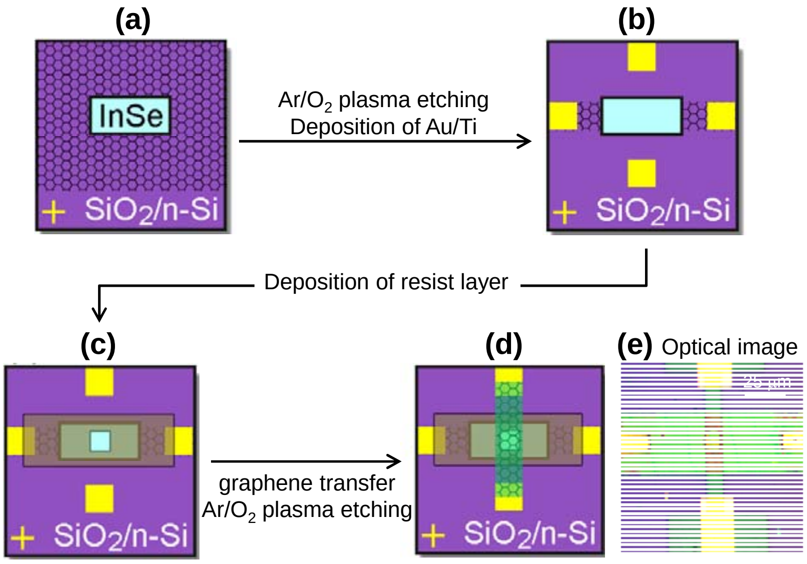

Figure S2. Main steps in the fabrication of graphene/*n*-InSe/graphene heterostructures using method B. **a)** An InSe flake is deposited on CVD graphene. **b)** The graphene layer is patterned and etched in an Ar/O_2 plasma to leave a graphene strip under the selected InSe flake. Au/Ti contact pads are then deposited at each end of the lower contact strip. **c)** An isolation layer, AR-

N, is formed to cover the InSe flake and the lower graphene contact. A window is formed in the AR-N layer on the top surface of the InSe. **d)** The top graphene contact strip is defined by etching in an Ar/O_2 plasma. The resulting upper contact runs at right angles to the lower graphene strip and overlaps the Au/Ti contacts formed at an earlier stage of the process. **e)** Optical image of a device (B_1) based on an InSe flake of thickness t = 130 nm.
